# A Review on the Beneficial Role of Silicon against Salinity in Non-Accumulator Crops: Tomato as a Model

**DOI:** 10.3390/biom10091284

**Published:** 2020-09-07

**Authors:** Jonas Hoffmann, Roberto Berni, Jean-Francois Hausman, Gea Guerriero

**Affiliations:** 1 Environmental Research and Innovation Department, Luxembourg Institute of Science and Technology, 5, rue Bommel, L-4940 Hautcharage, Luxembourg; jonas.hoffmann@list.lu (J.H.); jean-francois.hausman@list.lu (J.-F.H.); 2 Department of Life Sciences, University of Siena, via P.A. Mattioli 4, I-53100 Siena, Italy; roberto.berni@yahoo.com; 3 TERRA Teaching and Research Center, Gembloux Agro-Bio Tech, University of Liège, 5030 Gembloux, Belgium

**Keywords:** salt stress, cultivated tomato, silicon, biostimulants

## Abstract

Salinity is an abiotic stress that affects agriculture by severely impacting crop growth and, consequently, final yield. Considering that sea levels rise at an alarming rate of >3 mm per year, it is clear that salt stress constitutes a top-ranking threat to agriculture. Among the economically important crops that are sensitive to high salinity is tomato (*Solanum lycopersicum* L.), a cultivar that is more affected by salt stress than its wild counterparts. A strong body of evidence in the literature has proven the beneficial role of the quasi-essential metalloid silicon (Si), which increases the vigor and protects plants against (a)biotic stresses. This protection is realized by precipitating in the cell walls as opaline silica that constitutes a mechanical barrier to the entry of phytopathogens. With respect to Si accumulation, tomato is classified as a non-accumulator (an excluder), similarly to other members of the nightshade family, such as tobacco. Despite the low capacity of accumulating Si, when supplied to tomato plants, the metalloid improves growth under (a)biotic stress conditions, e.g., by enhancing the yield of fruits or by improving vegetative growth through the modulation of physiological parameters. In light of the benefits of Si in crop protection, the available literature data on the effects of this metalloid in mitigating salt stress in tomato are reviewed with a perspective on its use as a biostimulant, boosting the production of fruits as well as their post-harvest stability.

## 1. Introduction

Plants are sessile organisms and, therefore, they are continuously exposed to the surrounding environment. In order to carry out vital processes, such as photosynthesis, as well as vegetative and reproductive growth, plants need to adapt to the changing environment. They do so by activating a whole array of physiological processes, resulting from changes at the transcriptional and translational level [1,2], as well as from the activation of metabolic branches, leading to the synthesis of specialized metabolites [3,4,5].

Environmental conditions can be stable for a certain period, but they can also suddenly change. In this case, living organisms need to adapt to their new environmental conditions if these prevent them from achieving the successful completion of their life cycle [6]. Such unfavorable conditions are collectively referred to as environmental stresses.

Stress adaptation can take several aspects, depending on the type of altered environmental condition. If the stress is temporary and occurs suddenly, the plant will adapt to the new situation through non-permanent changes in its morphology or physiology. Therefore, these changes can be reversed if the environmental conditions return to normal and, in this case, acclimation occurs [7,8]. On the other hand, if the non-optimal conditions become common in a specific location, then plants need to adopt strategies to exploit their new environment in the most efficient manner and transmit these changes to their progeny. In this case, evolutionary adaptation takes place [9]. For example, some plant species can be specifically adapted to grow on volcano soils [10,11], littorals with high salinity conditions [12], or heavy metal-contaminated areas [13].

Stresses are generally divided into two different classes, depending on whether they entail the interaction with a living organism or not. The latter includes abiotic stresses that are caused by environmental changes, such as water stress, exposure to extreme temperatures, excess or lack of nutrients, high salinity, presence of heavy metals and ultraviolet radiation. Biotic stresses involve interactions with other organisms, such as another plant that will compete for nutrients and space, as well as animals and pathogenic bacteria or fungi, to name a few. Very often biotic and abiotic stresses can occur simultaneously: abiotic stresses tend to weaken plant’s defenses, making it more vulnerable to pests and pathogens. However, the simultaneous occurrence of abiotic and biotic stresses can increase the resistance to pathogens in plants [14].

Among the abiotic stresses, salinity deserves particular attention, in light of its deleterious effects on crop growth and yield [15]. The next paragraphs will treat (1) the problem of salt stress with the physiological impairments it causes to plants; (2) the response of cultivated tomato (*Solanum lycopersicum* L.) as an example of an economically relevant crop sensitive to excessive salinity; and (3) the mitigatory effects of silicon (Si) in *S. lycopersicum*, a Si non-accumulator.

## 2. Salinity Stress

Soil salinization is defined as the accumulation of salts in the soil solution that can be measured as the total dissolved solids (TDS) or the electrical conductivity [16], while sodicity is the predominance of Na^+^ ions that saturate the ion exchange sites in the soil instead of other ions, such as Mg^2+^ and Ca^2+^ [17]. Na^+^ ions damage soils because they disrupt the formation of macro-aggregates and promote colloidal dispersion, subsequently destroying the soil macro-structure.

Soil salinization has become a major concern for the past few years in the whole world since it is one of the consequences of climate change with the rise of the ocean’s level. Indeed, saltwater intrusion in groundwater dramatically increases the salt level present in soils, making them salty and unsuitable for agricultural production [18]. Coastal regions are most at risk, where it is estimated that sea levels rise at an average of 3.4 mm per year (https://ec.europa.eu/knowledge4policy/foresight/topic/climate-change-environmental-degradation_en). Additionally, soil salinization can increase because of storms and tsunamis that could directly flood soils.

Salinization can also occur in lands far from coastal regions due to several natural factors, such as the nature of the parent rock, or the irrigation ratio applied to soils and their evapotranspiration. The last factor is the main reason that causes arid and semi-arid soils to suffer from salinization. When there is not enough precipitation, the water rich in salts rises from the groundwater by capillarity, favoring the accumulation of salts in the upper layer of the soil, where they continually accumulate in the absence of precipitation. These natural events cause what is referred to as primary salinization, which is different from secondary salinization, determined, instead, by human intervention [19].

Irrigation is one of the major causes of soil salinization, especially in soils with high rates of evapotranspiration. This can be caused by irrigation with saline or brackish water. The problem can also occur when soils are irrigated with non-saline water or by rainfall with insufficient drainage conditions, or when watered in an inappropriate way, especially in areas with high evapotranspiration rates [20].

Some authors have attempted to model the salinity problem in the entire world, but because of inconsistencies in the available data, it was difficult to merge them to have a global overview of the situation, principally because of differences in soil classification among countries [21]. It was, however, estimated by the FAO in 2000 that 397 million ha were affected by salinity and 434 million ha by sodicity (http://www.fao.org/soils-portal/soil-management/management-of-some-problem-soils/salt-affected-soils/more-information-on-salt-affected-soils/en/). Unfortunately, more updated values do not exist for a worldwide overview [22].

To avoid salinization, irrigation has to be provided in sufficient amounts in order to leach the excess deposit of salts in the upper layers of the soil. However, irrigation water should be of good quality and leaching should be optimal. With respect to the first aspect, several costly techniques can be used for water desalinization or water recycling, such as inverted osmosis or electrodialysis; however, these are still in development [23].

Salts have two effects on plants. First, they play a role in water uptake due to the osmotic effect. Since soluble salts lower the osmotic potential, water is more difficult to be taken up by the roots. Therefore, plants must adopt special strategies to take up water from soil, despite its low water potential. The other effect of salts on plants is the potential toxic effect, especially for NaCl. Na^+^ is a competitor with other cations, such as Ca^2+^ and can compete for root cell wall binding sites. This causes disruption of essential physiological mechanisms, such as primary growth, by affecting pectin cross-linking [24]. At the cell wall level, such changes are reflected by a decreased stability and subsequent increase in rigidity. In maize leaves exposed to NaCl stress, the apoplast pH transiently alkalizes in response to chloride and triggers changes in protein abundances, notably related to the general phenylpropanoid pathway, hemicellulose biosynthesis and sucrose catabolism [25]. Notably, the transient alkalinization of the leaf apoplast causes a decreased extractability of free coumaric and ferulic acids, because they are cross-linked to hemicelluloses, resulting in an increased cell wall rigidification [25]. The increased rigidity of the cell wall is reflected by the reduced size of the leaves and roots. As an example, hemp (*Cannabis sativa* L.) leaves subjected to salt stress showed an increased expression of genes involved in lignification (phenylalanine ammonia lyase-*PAL* and cinnamyl alcohol dehydrogenase-*CAD*) and cellulose biosynthesis (secondary cell wall cellulose synthase genes-*CesA4*), a result indicating stiffening of the cell wall [26].

At the morphological levels, salt stress causes a reduced lumen size of xylem vessels within the plant’s conductive tissues: such an observation is linked to the decrease in cavitation, which is likely to occur at a higher frequency under salt stress [26].

**Figure 1:**
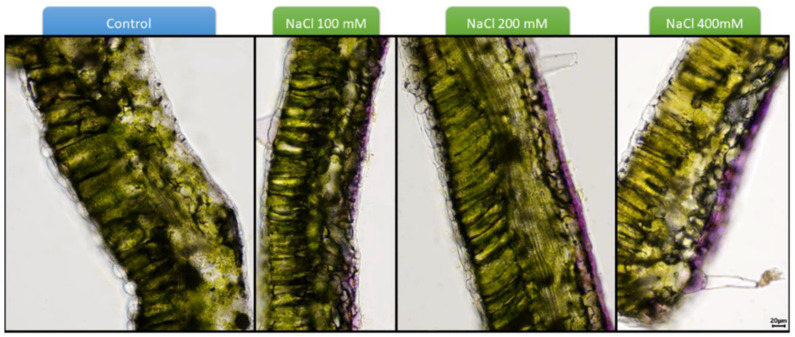
Vibratome transversal sections of tomato (*Solanum lycopersicum*, Micro-Tom cultivar) leaves exposed to increasing concentrations of NaCl and showing anthocyanins in the epidermal layer of the abaxial side. Bar: 20 µm (same magnification for all the pictures).

Salt stress causes oxidative stress that triggers, in its turn, the formation of reactive oxygen species (ROS), like the superoxide radicals responsible for DNA, RNA and protein oxidation. They also have an impact on lipids that constitute the cell membrane and can therefore compromise its composition and stability [27]. One of the responses of plants at the onset of salt stress is the production of antioxidant molecules, as well as enzymes scavenging ROS [4]. Examples of the former are phenolic compounds, whose chemical structure allows hydrogen atom transfer mechanisms (HAT) to occur via a pure H transfer, or an electron transfer followed by a proton release, or a proton loss followed by an electron transfer [28]. In *Cynara*, a plant mainly found in arid and semi-arid regions often subjected to salinity, NaCl stress induces the synthesis of phenolic substances in proportion to the increase in salt concentration and this increase in salinity has proven to be a good technique to enhance the secondary metabolite content in plants grown for nutraceutical use [29]. Among the most common secondary metabolites found at higher levels in plants stressed by salt are isoorientin, orientin, vitexin, rutin [30], as well as phenolic acids [31]. Anthocyanins often accumulate in the leaf epidermal cells of plants exposed to salt stress (Figure 1), where they are supposed to help decrease the osmotic potential by increasing the solute content within the cells [32]. However, it was shown that acyanic species achieved an osmotic adjustment that was similar to that of red-leafed evergreen species without synthesizing anthocyanins, thereby suggesting that these pigments are a small component of osmotic adjustment [33]. The metabolic investment necessary to synthesize anthocyanins and their abundance in some species suggest roles other than simple osmoprotectants [33].

Plants have different sensitivities to salt stress and are broadly classified into glycophytes and halophytes [34]: the former comprises species that are sensitive to salt stress, while the latter refers to salt-tolerant ones. The two groups are distinguished by specific physiological and biochemical mechanisms that, in the case of halophytes, make them resistant to high levels of salinity. For example, the halophyte *Salicornia dolichostachya* Moss is characterized by tonoplast H^+^ pumps whose activities are not increased by salt treatment, while the related glycophyte spinach (*Spinacia oleracea* L.) increased them instead [35].

Among the glycophytes of economic relevance, there is cultivated tomato, *S. lycopersicum*, which is affected by high salinity stress with consequent yield losses. In the next paragraph, the response of cultivated tomato to salt stress is discussed, with emphasis on the use of the cultivar Micro-Tom as an advantageous experimental model.

The different soil texture is also an important factor playing a central role on salt accumulation around the root zone and, consequently, on salt uptake by plants. Indeed, as reported by Li and co-authors, soils with textural layering can store more water than homogeneous soils (composed by a single layer) by hindering the vertical water movement during the process of infiltration (downwards) and evaporation (upwards) [36]. Thanks to their hydraulic properties, stratified soils are able to affect water dynamics with their thickness, composition and spatial organization of the inner layers: multi-layered soils can affect salt dynamics by diluting the salt ions’ concentration among the layers [36].

Zhai and co-authors have reported that straw mulching, combined with irrigation, significantly decreases the levels of salinity around the plant root-zone by creating a more favorable environment for the growth of tomato. More specifically, the application of a layer of organic material to the surface of soils (mulching) allows the vertical movement of salt from the root zone to the edge of the mulch [37].

In soils, cations such as Ca^2+^ and Mg^2+^ generate stable aggregates with the organic matter (i.e., with humic acids), which determine the soil properties, such as drainage and porosity. The high presence of sodium can replace other cations (Ca^2+^ and Mg^2+^), thereby altering the interaction with the organic matter and causing dispersion of soil particles. The application of gypsum (CaSO_4_) is considered to be a useful agronomical practice to replace and remove Na^+^ ions from the root zone [15].

Fertilizers and organic amendments are usually beneficial in mitigating the adverse effects of salinity, since salt stress inhibits the uptake of important ions, namely of K^+^, Ca^2+^ and NO_3_^−^ [38]. However, in a study conducted by Mori and colleagues, no major mitigating effects are observed on cherry tomato subjected to salinity and nitrogen fertilization [39]. Similarly, in another study, the yield of tomato under CaCl_2_, NaHCO_3_ and NH_4_^+^ fertilization remains unaffected in saline conditions, while glucose content and total soluble solids (TSS) are significantly higher [40].

Organo-mineral fertilizers composed of CaSO_4_, ground rice bran and humic acid are good alternatives to mitigate yield loss in tomato cultivated in saline soils, since proline, chlorophyll and antioxidant enzymes levels increase in stress conditions [38]. Therefore, combinations of organic and mineral fertilizers bring higher ameliorative effects in crops compared to the same fertilizers used individually. Indeed, as reported by Al-Yahyai and colleagues, higher yield and fruit quality are obtained by mixed fertilizers in tomato fruits [41].

## 3. Tomato as Model to Study the Response to Salt Stress

Cultivated tomato (*S. lycopersicum*) is a member of the Solanaceae family, which includes potato (*S. tuberosum*), pepper (*Capsicum annuum* L.), tobacco (*Nicotiana tabacum* L.) and petunia (*Petunia* sp.). It is an economically important plant largely cultivated worldwide, since its fruit is a fundamental constituent of the European diet and largely used in the preparation of several dishes. Its annual production is around 38 million tons and is principally located in Italy, Spain, Egypt and Turkey [42].

Tomato is easy to grow under laboratory conditions and produces a fleshy fruit (a berry) ideal for fruit-related studies, such as ripening, ethylene signaling and secondary metabolite production [43]. Tomato fruits are rich in antioxidants, notably carotenoids and vitamins (A and C) [44,45] and are thus important dietary sources of bioactive compounds. Compared to other model species, such as *Arabidopsis thaliana* and *Oryza sativa*, *S. lycopersicum* shows unique features: compound leaves, production of fleshy fruits, sympodial shoots [45,46] and a mostly indeterminate (vine) growth habit [45,47] (with some exception, see the Micro-Tom cultivar).

**Figure 2:**
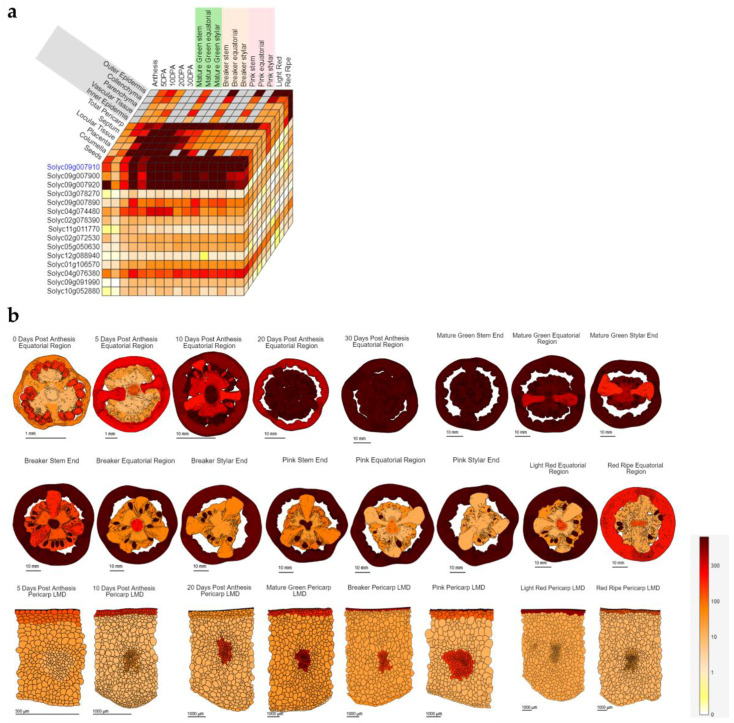
Example of Tomato Expression Atlas visualization using a phenylalanine ammonia lyase (*PAL*) gene (Solyc09g007910) as query. In (**a**) an expression cube with correlated genes; in (**b**) expression images of *PAL* in the fruit and its tissues at different developmental stages. The bar refers to the expression intensity in RPM (reads per million).

The entire genome of *S. lycopersicum* (the inbred cultivar “Heinz 1706”) was sequenced in 2012 [48], which allowed the development of a variety of bioinformatic tools for genomics/transcriptomics, proteomics and metabolomics. As an example, the Tomato Expression Atlas (TEA) developed by Fernandez-Pozo and colleagues [49] (available at http://tea.solgenomics.net/) is a web-based tool especially designed to display RNA-Seq data via an “Expression Cube”, which allows users to visualize and compare the expression profiles of multiple genes simultaneously. More specifically, it displays the expression pattern of the query transcript, together with highly correlated genes (Figure 2). It also allows visualizing data through different developmental stages of the fruit.

**Figure 3:**
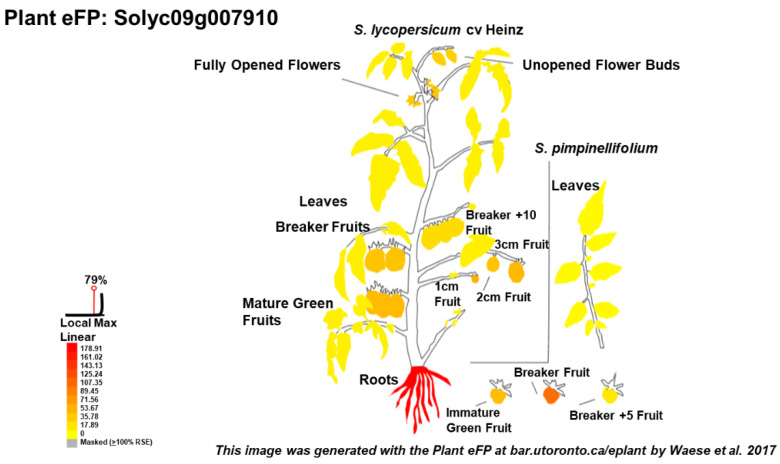
Example of an electronic Fluorescent Pictograph (eFP) visualization using the *PAL* gene Solyc09g007910 as query. The gene is highly expressed in the roots. The image was generated with the Plant eFP at bar.utoronto.ca/eplant [50]. Data are from [48] and are RPKM-normalized.

An eFP (electronic Fluorescent Pictograph) browser was developed by the university of Toronto [50] (available at http://bar.utoronto.ca/eplant_tomato/). It provides a visual presentation (a heatmap) of the different plant organs and the associated expression level of the target gene normalized by RPKM (reads per kilobase per million). This tool is therefore useful to investigate the expression in a defined plant organ and helps select the target genes in RT-qPCR studies. This approach is for instance useful to check the expression of genes of choice in a specific organ/tissue prior to the real experiment in the conditions/cultivars under investigation (Figure 3).

Secretom (available at https://solgenomics.net/secretom) is a database developed by the Solanaceae Genomics Network and dedicated to tomato cell wall proteomics where it is possible to access datasets obtained by, e.g., analyzing the glycoproteome of the tomato fruit, the secretome of the interaction between *Phytophthora infestans* and tomato, or the cuticular cell wall proteome.

Among the databases dedicated to tomato metabolomics, it is worth citing LycoCyc, developed by the Solanaceae Genomics Network (available at http://pathway.gramene.org/gramene/lycocyc.shtml) and the Metabolome Tomato database (MoTo DB) [51,52]. LycoCyc allows searching for a specific compound and then retrieves the information relative to the metabolic pathways and related compounds. Additionally, MoTo DB is an open access metabolite database dedicated to the tomato fruit and obtained by combining the literature and experimentally obtained data from cultivated, wild, as well as transgenic varieties.

Tomato is moderately tolerant to salinity [53] and, although studies have revealed that exogenous stresses (defined as “eustresses”) can increase the content of functional molecules in its fruits [54], severe salt stress is accompanied by losses in yield [55]. Domestication has reduced the salt tolerance in tomato: a recent genome-wide association study for the root Na^+^/K^+^ ratio in >360 tomato accessions has revealed that the highest differences were in a gene coding for a member of the HAK/KUP/KT (high-affinity K^+^/K^+^ uptake/K^+^ transporter) family [56]. Notably, knocking the gene out in tomato and the homologs in the distant species rice caused a salt-hypersensitive phenotype [56].

To increase the salinity tolerance in cultivated tomatoes, landraces are studied, which are more resistant to stresses and equally rich in functional molecules, i.e., antioxidants [44,57]. In this respect, non-commercial tomatoes, like the fruits from local plants, were shown to have higher contents of polyphenols and antioxidant compounds than a commercial counterpart [58].

Landraces, as well as ancient and wild varieties are valuable resources for breeding programs [58,59,60,61,62]. For example, wild relatives from the Galapagos Islands (*S. cheesmaniae* (L. Riley) Fosberg and *S. galapagense* S.C. Darwin and Peralta) are endemic species adapted to the highly saline coastal areas and a recent screening of 67 accessions revealed that different traits are involved in the high tolerance to salinity [62]. Tomato landraces from Southern Italy showed higher tolerance to drought via the constitutive increased activities of catalase, ascorbate peroxidase and glucose-6-phosphate dehydrogenase [63], while in the wild halophyte (*S. chilense*) exposed to NaCl, an increase in ethylene biosynthesis, accompanied by the upregulation of the 1-aminocyclopropane-1-carboxylic acid synthase (*ACCS2*) gene, was correlated with a high tolerance to salinity [64].

Among all the tomato cultivars, Micro-Tom is one of the most convenient for experimental investigations. It is a dwarf, bushy variety with a determinate growth habit, designed for ornamental purposes that differs from *S. lycopersicum* by three major dominant loci [65]. Although one of them, *dwarf*, is a brassinosteroid-related mutation, it was proven that Micro-Tom is a suitable model to address questions related to phytohormones, including brassinosteroids [66,67]. The frequency of the nucleotide mismatch in exons with the sequenced cultivar “Heinz 1706” was calculated to be 0.061% [68]. Because of its small size (10–20 cm in height) [69], reduced ground surface occupation, a rapid life cycle (70–90 days) and ability to be easily transformed [43,70], it allows high-throughput mutagenesis and routine experiments [43]. It grows at a high density under fluorescent lights and the space used to grow thale cress can be easily adapted to grow Micro-Tom plants [43]. Additionally, a database of Micro-Tom mutants, TOMATOMA [71] (available at http://tomatoma.nbrp.jp/about/aboutEn.jsp), was developed, which allows browsing of phenotypes and getting information on the metabolite content (amino acids, carotenoids and °Brix). With respect to salt stress, Micro-Tom was shown to accumulate phenolic compounds in its leaves [72], a result witnessing its suitability to address questions related with the antioxidant response as a consequence of abiotic stresses. Last, but not least, Micro-Tom is also suitable to study the interaction with plant growth-promoting bacteria; indeed, a study showed that a bacterium of the genus *Streptomyces* isolated from the peanut rhizosphere was capable of alleviating the stress symptoms in salt-stressed Micro-Tom [73].

## 4. The Protective Role of Si against Salinity: The Case of Cultivated Tomato

With respect to Si accumulation, plants are usually classified into accumulators (e.g., rice and horsetail), intermediate-types (nettle and cucumber) and excluders (or non-accumulators, e.g., members of the nightshade family). Although classified as an excluder, a homolog of the rice aquaporin *Lsi1* mediating the entry of Si was recently isolated and characterized in tomato and found to be functional via expression in rice *lsi1* mutants [74]. When overexpressed in tomato, a higher accumulation of Si was observed in the roots and root cell sap, but not in the shoots. This finding contradicts previous results showing that the aquaporin was not capable of mediating the entry of Si because of a spacing of 109 amino acids between the asparagine-proline-alanine (NPA) motifs, instead of 108 required for permeability to the metalloid [75]. It was concluded that what makes tomato an excluder with respect to Si is the absence of a functional Si efflux transporter Lsi2 [74]. Despite the classification into the excluders’ category, tomato shows amelioration of stress symptoms when supplemented with Si and silica nanoparticles (N-SiO_2_). For example, Si together with salicylic acid activated the antioxidant systems of tomatoes stressed by a high pH (by, e.g., upregulating the genes peroxidase, ascorbate peroxidase, superoxide dismutase and catalase) and decreased the concentration of abscisic acid in the shoots and roots [76]. Interestingly, in germinating tomato seeds exposed to drought, Si supplementation contributed to decrease the activity of peroxidase, while increasing that of superoxide dismutase and catalase [77]. Under water stress, Si contributed to lower the decrease in chlorophyll and carotenoids and improved photosynthetic parameters (PSII maximum photochemical efficiency, photosynthetic electron transport rate and upregulation of photosynthesis-related genes) [78]. Additionally, Si ameliorated the response of tomato against the plant pathogenic bacterium *Ralstonia solanacearum* by stimulating, at the gene level, pathogen-associated molecular pattern-triggered immunity, resistance to oxidation and water-deficits and by increasing the content of lignin-thioglycolic acid, which strengthens the cell walls of roots [79].

Under salt stress (150 mM NaCl), Si (2 mM Na_2_SiO_3_) increased the content of K, Ca and Mg and decreased that of Na and Cl in tomato roots, stems and leaves. This was not mediated by a reduced translocation from root to stem or stem to leaves, but to a salt dilution effect triggered by an improved growth, i.e., a higher shoot biomass accumulated under salt stress and Si application [80]. A previous study showed that the metalloid mitigated the loss in dry biomass in salt-stressed tomato plants. It also proved that leaf turgor potential improved in the presence of Si [81]. This last observation is due to the precipitation of Si as opaline silica in the cell walls of epidermal cells, which creates a layer hindering water loss under abiotic stress.

Cao and colleagues investigated the impact of Si on tomatoes subjected to drought stress under PEG treatment for 12 days and found that the metalloid alleviated the negative effect of PEG by increasing significantly the root absorbing surface area and promoting radial hydraulic conductivity through a decrease in the cortex-to-root diameter ratio [82]. Proline, soluble proteins and the sugar content of roots were also higher, with a peak reached at the beginning of stress application in Si-treated plants, while the O_2_^−^ and H_2_O_2_ production were lower. Additionally, Si application delayed the decline of SOD and CAT activities compared to stressed plants.

Results concerning the protective impact of Si on the photosynthetic machinery were obtained by Muneer and colleagues who performed a proteome analysis on tomato chloroplasts after supplying high concentrations of Si (2.5 mM), which is beyond the solubility limit [83]. They observed a positive effect of Si supplementation on oxidative damage, chlorophyll content and photosynthesis, especially at higher salinity (50 mM). Proteomics showed an increased amount of PSI and PSII complexes, as well as of cytochrome *b6/f* and ATP-synthase in Si-supplemented plants, compared to non-supplemented, salt-stressed plants.

The beneficial effects of Si application were also studied in relation with the post-harvest stability of tomato fruits obtained from plants cultivated in moderate salinity conditions (50 mM) and treated or not with potassium silicate, 2 mM [84]. In particular, Costan and co-authors supplied further Si by dipping tomato fruits in a sodium silicate solution (5000 mg L^−1^) and then they compared different parameters, such as TSS, weight and total phenolic content (TPC), against control fruits submerged in a dH_2_O solution, after 15 days of storage [84]. The authors showed a 42% increase in TSS and weight in the treated fruits as compared to the control ones, as well as a decrease in TPC in control fruits equal to 37%. Interestingly, fruits obtained from plants supplemented with Si and not dipped in sodium silicate also showed improvements in their qualitative parameters, in particular in fruit firmness [84].

The results shown therefore confirm that Si fertilization can alleviate the harmful effects of high salinity in tomato plants and prove that the post-harvest treatment with this metalloid is an interesting approach to increase the shelf-life of the fruits, while maintaining the qualitative parameters.

The constant search for new methods to improve crop yield and to alleviate the negative effect of stresses in plants has motivated the experimentation of new technologies in agriculture, for example nanotechnologies. A strong body of evidence in the literature has proven the beneficial roles of nanoparticle applications on plants, especially in increasing the yield and quality of crops [85]. The peculiar nanoscale size allows these materials to have a large surface area, as well as a high solubility and reactivity [86,87]. Thanks to these features, nanoparticles are able to penetrate in leaves and roots by different ways, such as through the cuticle as well as the stomata symplastic and apoplastic pathways [88]. When used as fertilizers, nanomaterials can act as carriers by encapsulating nutrients directly or these can be coated with a polymer film [89].

Concerning tomato plants, different studies have shown the use of nanofertilizers to improve their growth and yield. For instance, Shankramma and co-authors tested fertilizers enriched with iron oxide nanoparticles and showed a general improvement in growth parameters, in particular in seed germination, as well as in root and shoot lengths [90]. Marchiol and co-workers proposed hydroxyapatite nanoparticles (N-HA) as an innovative tomato fertilizer, showing a strong boost in root elongation together with non-toxic effects [91].

With respect to Si, Haghighi and Pessarakli tested both Si (as silicate) and N-SiO_2_ on cherry tomato plants exposed to different salt concentrations (i.e., 0, 25 and 50 mM) and observed that Si and N-SiO_2_ significantly increased the stomata conductance, growth, water uptake and root volume in plants exposed to salinity [92]. Although N-SiO_2_ application did not show substantial differences as compared to Si, these particles are interesting because they show the properties of nano-materials and the beneficial effects of Si. González-Moscoso and co-authors studied the positive actions of N-SiO_2_ in alleviating the deleterious effects of arsenic at different concentrations on tomato plants. The authors observed a decrease in the oxidation-reduction potential (ORP), corresponding to a higher antioxidant potential in plants treated with N-SiO_2_ than in the control plants [93].

Due to the proven role of Si in plant protection against a wide range of exogenous stresses, the use of N-SiO_2_ can provide an effective way to improve crops’ performance [94], especially under a changing environment.

Si fertilization may bring beneficial effects in tropical and subtropical areas where leaching and intensive crop cultivation practices cause depletion of Si from soils. Therefore, this type of fertilization could represent a source of Si able to support poor soils, where the insufficiency of Si may be a limiting factor, contributing to lower yields [95].

In soilless cultures, Si enrichment of irrigation solutions was proven to be beneficial by Jarosz and colleagues, who showed an increase in fruit yield and a higher content of Si in leaves [96].

As previously reported by Wang and Xing, finding the optimal combination between irrigation and fertilization may allow a significant increase in yield in tomato cultures [97]. The authors showed that a higher fruiting rate can be achieved by a better combination of the temporal and spatial distribution of water and nutrients during plant growth.

Taking into account these pieces of evidence, the optimal combination of Si fertilization, mulching and spatial/temporal mineral and organic fertilization will represent a step forward towards contrasting the excessive salinity in different soils and improve the yield of tomato cultivars.

## 5. Future Perspectives

The use of Si in agriculture practices has increased in the last years, due to its proven beneficial effect on crop yield, as well as by alleviating the negative effects caused by different stresses, such as high salinity or drought [98]. Si is included in the category of biostimulants [99,100]. Its application can be particularly interesting in combination with eustress [54]; for example, on crops growing on mildly saline soils. Mild salinity can act as eustressor, contributing to boost the production of functional molecules in fruits. Si can also provide a sustainable approach to preserve good yields and biomass production under mild salinity. Additionally, it will be interesting to study the effects of Si fertilization on the growth and yield of non-commercial ancient varieties. Indeed, some studies in the literature have demonstrated the interesting nutraceutical potential of such plants, which can thrive in wild environments [59]. For example, non-commercial varieties of tomatoes cultivated in Italy were shown to have higher contents of antioxidant compounds with respect to commercial ones [58,60]. By growing in the wild and, therefore, being continuously exposed to (a)biotic stressors, the yield of such plants cannot meet the demands of the market. However, Si fertilization can be used as a sustainable practice in regional agricultural programs aimed at valorizing such non-commercial varieties, to increase their yield and for the manufacture of products with enhanced organoleptic and nutraceutical characteristics.
